# AGREEMENT BETWEEN MEASURED AND PERCEIVED NUTRITIONAL STATUS REPORTED BY PRESCHOOL CHILDREN’S MOTHERS

**DOI:** 10.1590/1984-0462/;2017;35;3;00011

**Published:** 2017-07-31

**Authors:** Dixis Figueroa Pedraza, Carolina Pereira da Cunha Sousa, Ricardo Alves de Olinda

**Affiliations:** a Universidade Estadual da Paraíba, Campina Grande, PB, Brasil.

**Keywords:** Weight perception, Overweight, Nutritional status, Child, Daycare centers

## Abstract

**Objective:**

To verify the agreement between nutritional status perceived by mothers and that diagnosed in preschool children, by providing the differences according to children’s sex and age.

**Methods:**

Study with data from a cohort of 269 preschool children assisted in public daycare centers of Campina Grande, Paraíba (Northeast Brazil). Children’s information about their date of birth, sex and nutritional status (weight/stature Z scores) was collected. Furthermore, the mothers were asked about their perception of children’s weight. The diagnostic agreement between the measured nutritional status and that perceived by mothers was assessed through the weighted Kappa test, with a 5% significance level.

**Results:**

The percentage of disagreement between the measured nutritional status and that perceived by mothers was 32.7%, with Kappa of 0.122, which is considered insignificant. There was a remarkable overweight underestimation (69.6%). Agreement between maternal perception of overweight and the diagnosed nutritional status was higher for older children (36-59 months *versus* 24-35 months) and for girls.

**Conclusions:**

The study regarding maternal perception of preschool children’s nutritional status showed the difficulty that mothers face in recognizing the real nutritional status of their children, especially the underestimation of overweight. Maternal perception of overweight in children is misrepresented in boys and in younger children with more importance.

## RESUMO

**Objetivo:**

Verificar a concordância entre o estado nutricional percebido pelas mães e o diagnosticado em crianças pré-escolares, distinguindo diferenças segundo o sexo e a idade da criança.

**Métodos:**

Estudo com dados provenientes de uma coorte de 269 crianças pré-escolares assistidas em creches públicas de Campina Grande, Paraíba. Coletaram-se informações sobre a data de nascimento, sexo e estado nutricional das crianças (escores-Z de peso/estatura). Além disso, as mães foram questionadas em relação à percepção sobre o peso da criança. A concordância diagnóstica entre o estado nutricional aferido e o percebido pelas mães foi avaliada pelo teste Kappa ponderado, com significância de 5%.

**Resultados:**

O percentual de desacertos entre o estado nutricional percebido pelas mães e o diagnosticado foi de 32,7%, com Kappa de 0,122, considerado desprezível. Destacou-se, principalmente, o excesso de peso subestimado (69,6%). A percepção materna do sobrepeso e o diagnosticado apresentou maior concordância nas crianças de maior faixa etária (36-59 meses *versus* 24-35 meses) e nas meninas.

**Conclusões:**

O estudo da percepção materna do estado nutricional de crianças pré-escolares mostrou a dificuldade que as mães apresentam em reconhecer o real estado nutricional de seus filhos, principalmente a subestimação do sobrepeso. A percepção materna do sobrepeso das crianças é mais distorcida em meninos e nas crianças mais novas.

## INTRODUCTION

Body image is understood as a multidimensional construction that describes the internal representation of body structure and physical appearance, which is formed not only by the mind, but also by feelings associated with the representation of one’s self.^1^ ^,^ ^2^ It is a process that happens throughout life, and its structure is easy in the first years of development, due to physiological, affective, and social conditions.^3^

Body self-perception takes place as the child develops and acquires awareness of his/her body characteristics; therefore, the child can recognize his/her image in the mirror only by the time he/she is 2 years old.^4^ ^,^ ^5^ Thus, the parent’s perception over their children’s nutritional status is relevant for actions to prevent nutritional disorders and for repercussions regarding body image.^4^

Hence, it is noteworthy that good parental relationships are important in the construction of a positive body image^6^ and of the mother figure as a regulator of children’s food behavior, preferences, and standards.^7^ The misrepresented maternal perception or the non-recognition of the children’s nutritional status may cause the onset of physical problems and psychological disorders in the child.^2^ Children’s dissatisfaction with their own body, in turn, may be associated with the child’s maternal perception in childhood, which can make them have harmful behaviors regarding their nutritional status and health.^8^

Parental perception studies regarding their children’s nutritional status show a trend of maternal underestimation of weight excess in overweight or obese children, by not recognizing them as such,^9^ ^,^ ^10^ which can therefore impede the adoption of preventive measures, neglect the search for specialized professional support, and damage adherence to the treatment recommended in positive cases, thus discouraging lifestyle modifications.^2^ ^,^ ^7^ ^,^ ^9^ This situation has also been found in Brazilian studies.^9^ However, only few studies have used the reference standard recommended by the World Health Organization (WHO)^10^ in their evaluations to diagnose nutritional status, whose use allows early recognition of overweight children and includes breastfed Brazilian children.^11^ Furthermore, few are the studies developed in Brazil including children who attend daycare centers,^9^ despite the possible behavioral changes due to the school environment.^10^ Thus, the present study aimed to verify the agreement between the nutritional status perceived by mothers and that diagnosed in preschool children, by providing differences according to the children’s sex and age.

## METHOD

This study is part of a cohort comprised of data collection in two different moments, with a 12-month interval between the first (from October to November 2011) and the second observation (from October to November 2012). Data collection occurred in public daycare centers of Campina Grande, Paraíba State, Brazil, which belong to the Department of Education.

The city of Campina Grande is located in the Rural Region of Paraíba, 120 km away from João Pessoa - the State capital. It has a territorial area of 671 km^2^ and a population of 402,912 residents (current estimations). Because of its privileged geographic position, Campina Grande is a region of convergence with approximately 232 cities from Paraíba State and neighboring States. Its residents have to dislocate for the city to find the provided services, like health services.

During data collection in the first observation, 25 public daycare centers were in operation in different neighborhoods of the city, located in poor areas. Based on the location, 23 daycare centers were in the urban area and two in the rural area. According to the age range, only 8 daycare centers worked as nurseries (for children aged 4 to 20 months) and 93% of the children were 24 months old or older. Children remained in the daycare center for 8 hours on a daily basis or for only 4 hours per day, with equivalent amount of daily meals.

The population eligible to participate in the study included children registered and attending daycare centers (2,749, of whom 199 are in nurseries), except for twins, adopted children, whose mothers were aged less than 18 years and with physical disabilities that provided more difficulties in the anthropometric assessment, which led to 166 exclusions. In the case of siblings, one of them was chosen for the study in the daycare centers. Because the minimum age of children was 12 months in the first visit, those children aged less than 24 months were not included in the present study, considering that in the second moment all children were aged 2 years old or older.

In the first step, a proper sampling size was estimated based on the procedure for proportion description.^12^ An estimated overweight prevalence (*p*) of 7.0% in children younger than 5 years old, according to the Brazilian National Survey of Demography and Health,^13^ a sampling error (d) of 3 percentage points and a 95% confidence level (Zα^2^ = 1.96^2^) were considered. There was a 10% increase in the calculated value (252) for losses and refusals, and a sampling design effect of 1.2; which resulted in a sample of 335 subjects. Proportional sampling sizes were considered for the children’s study according to the daycare center location area (urban, rural) and child’s age (less than 2 years old, 2 years old or more).

For sample selection, 14 daycare centers were chosen through simple randomization, which represents all city neighborhoods with working daycare centers. One daycare center was chosen of those located in the rural area and two of those with nursery services. Subsequently, on the possession of a list regarding children assisted in daycare centers, 15 children aged 24 months or older were systematically chosen by small-sized daycare centers (3 daycare centers); 20 middle-sized daycare centers (3 daycare centers); 25 large-sized daycare centers (5 daycare centers); and 35 of a chosen daycare center from the rural area. In each chosen daycare center with nursery services, 35 children aged less than 2 years were chosen.

In the second observation, aiming at decreasing losses, three attempts of visits (one in the daycare center and two at the household) were conducted. The mothers were encouraged to participate through the development of activities, like returning the results of the children’s nutritional status evaluation performed in the first visit and educational actions on feeding, nutrition, and infectious diseases.

Duly trained, standardized and supervised interviewers (students and health professionals) collected information for the present study both in the first and second observations. In both occasions, the mothers answered a questionnaire regarding their children’s health status and nutrition. In the first observation, the questionnaire was applied in the daycare centers; whereas in the second, it was used in daycare centers or at the household. For this study, in the first moment, information regarding children’s date of birth and sex, from the Child’s Health Notebook, was used. The children’s age was calculated in months by the difference between the date of the first interview and the date of birth. In addition, information regarding the maternal perception of the child’s weight and the anthropometric, weight and height variables obtained in a second moment were used. In order to obtain the perception, the mother was asked without further explanation what she believed the child’s current weight was, with three options of answers in the questionnaire: below weight, within weight or overweight.

Children had their heights measured with a stadiometer (WCS^®^, Biogênese Comércio de Artigos Médicos Ltda., Curitiba, Paraná, Brazil), with a 200 cm amplitude and 0.1 cm subdivisions. All children were weighted using the electronic weighing platform scale with capacity for 150 kg and graduation in 100 g (Tanita UM-080•, Tanita Corporation, Tokyo, Japan). The measurements were taken in duplicate, always in the daycare centers, in the presence of the mother, by accepting a maximum variation of 0.3 mm for height measurement and 0.1 kg for weight measurement. If these limits were eventually exceeded, the measurement was repeated by writing down two measurements with closer values and using their mean for registration purposes. A technical measurement error equal to or lower than 2.0 was acceptable. Trained anthropometrists took the measurements according to standardized technical rules and following the procedures recommended by the WHO.^14^

The children’s weight/height Z scores were calculated by means of the WHO Anthro 2009 (World Health Organization, Geneva, Switzerland) program. The population of the Multicentre Growth Reference Study was a reference, since it is currently recommended by the WHO.^15^ Children with Z scores lower than -2 standard deviations of the reference population were considered low weight; whereas those with scores higher than +2^14^ were overweight.

The agreement between the maternal perception of their children’s nutritional status (eutrophic, overweight, low weight) and the diagnosed status according to the WHO ranking (eutrophic, overweight, low weight) was evaluated using the weighted Kappa test, with a 5% significance level. The analysis of results was performed for the total sample by sex (male, female) and age (24-35 months, 36-59 months). Kappa values were interpreted based on the criteria proposed by Landis and Koch,^16^ whose strength of agreement is divided into: no concordance (< 0.00); insignificant (0.00-0.20); mild (0.21-0.40); moderate (0.41-0.60); large (0.61-0.80); and excellent (0.81-1.00).

The Research Ethics Committee of *Universidade Estadual da Paraíba* approved the project under No. 0050.0133.000-11. All mothers whose children were evaluated and the daycare centers’ principals signed the Free Informed Consent Form.

## RESULTS

**Figure 1:**
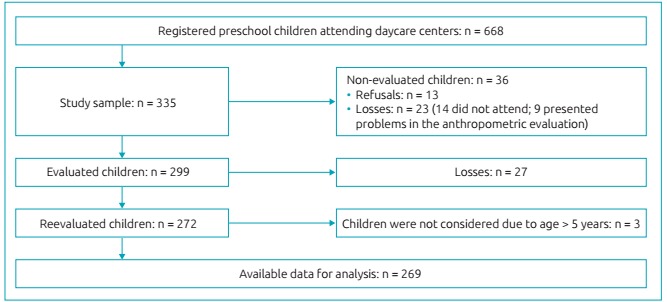
Flowchart of the study population/sample selection.

During the first study moment, of the 335 children, 13 mothers refused to take part in the research; 14 did not attend the daycare center or were not with the mother during the day of data collection; and it was impossible to perform the anthropometric evaluation in 9 of them, (unease or crying children), which results in a study of 299 children. In the second moment, after the loss of 27 children, 272 could be reassessed (all of them were aged 2 or older, because during the first visit, the children’s minimum age was 12 months). The three children who were older than 5 years during the second visit were not included and, therefore, the data analysis corresponded to a total of 269 children in this study (Figure 1).

Among the 269 children studied, 54.3% were male and 52.4% were aged 36-59 months. As observed, eutrophic was the most common nutritional status (86.6%), followed by overweight (8.6%). 4.8% of the children had low weight (Table 1). Evaluated and lost children did not have different characteristics.

**Table 1:**
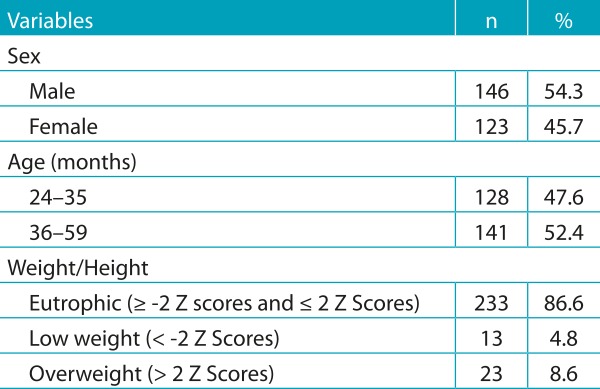
Characteristics of children attending public daycare centers of Campina Grande, Paraíba, 2011.

The mothers classified 70.6% of the children with the correct weight; 25.3%, below weight; and 4.1%, overweight. The percentage of errors between the mother’s perceived nutritional status and the diagnosed weight observed in the evaluated population was of 32.7%; therefore, the mother noticed one in every three children with a wrong perception. The agreement between the maternal perception of the child’s nutritional status and that diagnosed measured through Kappa test was 0.122 (*p*=0.02), which was insignificant according to the adopted classification. For overweight children, a 0.377 Kappa value (*p*<0.01) was obtained and represents a mild agreement (Table 2).

**Table 2:**
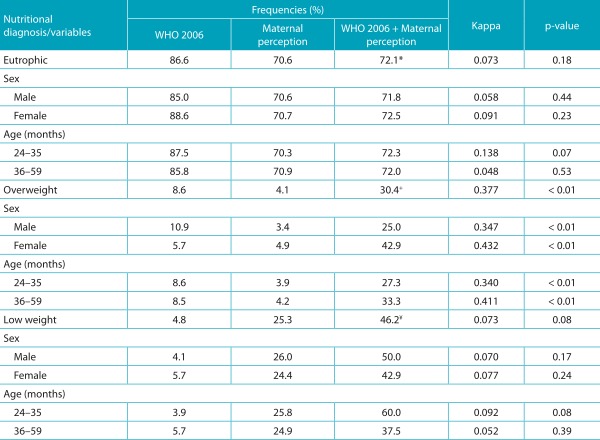
Nutritional diagnosis frequency of preschool children (%) and diagnostic agreement (Kappa) considering the maternal perception and the nutritional status according to weight/height of children attending public daycare centers of Campina Grande, Paraíba, 2011.

WHO: World Health Organization. *The mothers reported that children with low weight and overweight, 53.8 and 65.2% respectively, were eutrophic. ^+^The mothers noticed overweight in 1.7% of the eutrophic children. ^¥^The mothers noticed low weight in 26.2% of the eutrophic children and 4.4% of the overweight children.

According to the classifications of the perceived and diagnosed nutritional status (Table 2), the mothers considered most of the children to have proper weight (72.1%). In overweight children and in those with low weight, the correct answers corresponded to 30.4 and 46.2% respectively. It is noteworthy that among children with low weight and overweight, the mothers reported 53.8 and 65.2% as eutrophic, respectively. Only 25.0% of overweight boys had their nutritional status correctly identified by the mothers, and the prevalence of correct answers among girls was 42.9%. Among children aged 36-59 months, the mothers identified 33.3% correctly with overweight, whereas only 27.3% of those children aged 24-35 months had the same result. An agreement between the maternal perception and the diagnosed perception in overweight children, both for children from the total sample and for sex and age range, was considered. According to sex, a moderate agreement was seen among girls and mild among boys. Based on age, there was an agreement of moderate intensity among children aged 36-59 months and mild among the youngest.

## DISCUSSION

The nutritional status evaluation of children from the present study pointed out to convergent results for national range, like the 2006 Brazilian National Survey of Demography and Health of Child and Women,^13^ which presented high prevalence of weight excess and lower percentages of low weight. Similar results were also found, for instance, in a population-based study carried out in cities from Maranhão^17^ State, in which the authors suggest a children’s nutritional transition process with predominance of weight excess.

This result becomes even more disturbing considering that the mothers from this study presented a distorted perception of their children’s nutritional status, mainly in overweight ones. Such fact has been mentioned in the literature and emphasized, due to its implications in the adoption of health promotion and prevention actions.^2^ ^,^ ^4^ ^,^ ^10^ ^,^ ^18^ The difficulty of mothers to identify their children’s proper weight, especially overweight, reinforces the importance of increasing monitoring in health promotion since the first years of life as part of health primary care actions.^4^

Underestimation of child’s weight excess by the mothers is a recurring fact in the literature,^2^ ^,^ ^10^ ^,^ ^18^ ^,^ ^19^ ^,^ ^20^ ^,^ ^21^ ^,^ ^22^ ^,^ ^23^ which is similar to the maternal perception, in this paper, of overweight children considered as euthrophic. Two systematic reviews in the literature on the theme reached the same conclusion with the same line of reasoning;^9^ ^,^ ^24^ emphasis should be given on the context of the present paper for a higher trend of this outcome in children aged 2-6 years,^24^ which is in accordance with that reported herein. The importance of this study is in the fact that such outcome was found among children who attend daycare centers, who may present some specificities caused by institutionalization, with shared responsibility and different perspectives that might influence their care.^25^

It is noteworthy the possible relation of this underestimation with the trend of increasing weight excess - a phenomenon that can misrepresent the perception of normality standard (children with weight excess reported as having normal weight and eutrophic children as low weight).^10^ ^,^ ^19^ ^,^ ^20^ Many mothers also believe that excess weight will distribute homogeneously after the child gets older, so he/she will not become an overweight adolescent.^21^ ^,^ ^26^ The relevance of these results causes the influence of maternal perception of the child’s nutritional status in overweight prevention and treatment.^9^ ^,^ ^18^ ^,^ ^19^ ^,^ ^22^ ^,^ ^24^ Hence, it is important to know about children’s obesity and its risks for health, which can be created by means of educational practices.^27^ The agreement observed for overweight children might be associated with perceptible clinical signs of such conditions, or with its recognition as a sickening condition.^9^ ^,^ ^27^ Regardless of the adopted reference curve, results are similar to those found for preschoolers from Portugal.^2^ The use of the new WHO growth curves is suggested not only for enabling a proper diagnosis of overweight and obesity, but also for allowing the professionals to use them to assist parents in the understanding of the real conception of their children’s nutritional status. Lack of child’s real weight perception represents the main factor that has a negative influence on the parents’ behavior with treatment of weight excess.^20^ ^,^ ^24^ ^,^ ^28^

A study developed with children from public and private daycare centers in Balneário Camboriú, Santa Catarina, observed that only 17.4% of the boys with excess weight were correctly identified by their mothers; and the prevalence of agreement among girls was of 52.2%.^10^ These results are in agreement with those found herein; therefore, they show a moderate agreement between the maternal perception of overweight and that diagnosed for girls, whereas such agreement is mild for boys. Some researchers have reported a higher maternal concern and sensitivity with excessive weight gain in girls, which shows an underestimation of weight among boys and an overestimation among girls. This is mainly due to the larger attention that society gives to female body image, which causes higher levels of body dissatisfaction in women than in men.^4^ ^,^ ^6^ ^,^ ^21^ ^,^ ^23^ Therefore, as mentioned before,^8^ preventive measures regarding body dissatisfaction should be conducted for each sex independently.

In addition, the present study results are also remarkable for the higher agreement between maternal perception of the nutritional status and that diagnosed for older overweight children (36-59 months), which follows the results of previous and similar investigations.^4^ ^,^ ^7^ The maternal concern with children’s overweight is more evident with increasing age or when the child experiences negative effects associated with excess weight, such as reduced physical mobility, use of clothes with proportional size to those of adults, and reduced self-esteem - more prevalent in older ages.^4^ ^,^ ^7^

The verbal scale used in such study constitutes the most used instrument to assess the maternal perception of their children’s nutritional status, which allows the comparison of the proposed objective accomplishment. Furthermore, the use of new WHO growth curves as a reference standard, because they are based on breastfed children, allows earlier recognition of children with excess weight ^10^ - with importance conditioned to the lack of studies with these characteristics and regarding child’s overweight prevention. Despite this, verbal evaluation predisposes to a higher distortion of children’s nutritional status perception than the visual scale comprised of pictures of shapes^7^ ^,^ ^9^ ^,^ ^29^ - an aspect that should be considered when interpreting the current study results. However, the fact that mothers underestimated their children’s nutritional status regardless of the kind of scale used to evaluate their perception is also a highlight.^30^ Hence, the results seemed to be influenced by emotional aspects and other variables like age and educational level, which might cause damage to their children’s weight maternal ranking;^7^ ^,^ ^9^ ^,^ ^29^ however, it was not the scope of this study.

This study presents other limitations regarding the inclusion of a few variables, which interferes with a better understanding pf the factors involved in the maternal perception error of a child’s nutritional status. The nutritional status perception analysis should include a large variety of conditions, such as socioeconomic aspects, which would enable identifying other risk groups for the body image distortion. In addition, qualitative methods would be necessary to explain the influence of some questions, such as cultural standards.

In conclusion, the study of maternal perception of preschool children’s nutritional status showed the difficulties that mother face to recognizr their children’s real nutritional status, mainly the underestimation of overweight children. The maternal perception of overweight children is more misrepresented in boys and in younger children. These results are important pieces of evidence because the proper maternal perception of the child’s nutritional status, especially of weight excess, might predispose to treatment adherence. These circumstances might also reflect positively in the prevention of possible body image disorders, which are the result of the socially accepted and valued thinness standard considered as ideal. All these factors should be considered with special caution, because they present relevant repercussions in health promotion practices.
